# Effects of Carbamazepine, Lacosamide and Zonisamide on Gliotransmitter Release Associated with Activated Astroglial Hemichannels

**DOI:** 10.3390/ph13060117

**Published:** 2020-06-05

**Authors:** Kouji Fukuyama, Yuto Ueda, Motohiro Okada

**Affiliations:** Department of Neuropsychiatry, Division of Neuroscience, Graduate School of Medicine, Mie University, Tsu, Mie 514-8507, Japan; k-fukuyama@clin.medic.mie-u.ac.jp (K.F.); uedayuto@gmail.com (Y.U.)

**Keywords:** astrocyte, carbamazepine, lacosamide, zonisamide, microdialysis, hemichannel

## Abstract

Recent studies using the genetic partial epilepsy model have demonstrated that hyperfunction of astroglial hemichannels contributes to pathomechanism of epileptic seizure. Therefore, to explore the novel anticonvulsive mechanisms, the present study determined the effects of voltage-dependent Na^+^ channel (VDSC)-inhibiting anticonvulsants, carbamazepine (CBZ), lacosamide (LCM), and zonisamide (ZNS) on the astroglial release of l-glutamate and adenosine triphosphate (ATP). The effects of subchronic administration of therapeutic-relevant dose of three anticonvulsants on the release of l-glutamate and ATP in the orbitofrontal cortex (OFC) were determined using microdialysis. The concentration-dependent effects of acute and subchronic administrations of anticonvulsants on astroglial gliotransmitter release were determined using primary cultured astrocytes. The concentration-dependent effects of subchronic administrations of anticonvulsants on connexin43 (Cx43) expression in the plasma membrane of primary cultured astrocytes were determined using the Simple Western system. An increase in the levels of extracellular K^+^ resulted in a concentration-dependent increase in the astroglial release of l-glutamate and ATP. The depleted levels of extracellular Ca^2+^ alone did not affect astroglial gliotransmitter release but did accelerate K^+^-evoked gliotransmitter release via activation of astroglial hemichannels. Both non-selective hemichannel inhibitor carbenoxolone (CBX) and selective Cx43 inhibitor GAP19 prevented both gliotransmitter release through activated astroglial hemichannels and the hemichannel-activating process induced by elevation of the levels of extracellular K^+^ with depletion of the levels of extracellular Ca^2+^. ZNS subchronically decreased Cx43 expression and acutely/subchronically inhibited Cx43 hemichannel activity. LCM acutely inhibited hemichannel activity but did not subchronically affect Cx43 expression. Therapeutic-relevant concentration of CBZ did not affect hemichannel activity or Cx43 expression, but supratherapeutic concentration of CBZ decreased Cx43 expression and hemichannel activity. Therefore, the present study demonstrated the distinct effects of CBZ, LCM, and ZNS on gliotransmitter release via modulation of astroglial hemichannel function. The different features of the effects of three VDSC-inhibiting anticonvulsants on astroglial transmission associated with hemichannels, at least partially, possibly contributing to the formation of the properties of these three anticonvulsants, including the antiepileptic spectrum and adverse effects regarding mood and cognitive disturbance.

## 1. Introduction

The principle antiepileptic mechanism of many first-line anticonvulsants against partial epilepsy, including carbamazepine (CBZ), lacosamide (LCM), and zonisamide (ZNS), is considered to be use-dependent inhibition of voltage-dependent Na^+^ channel (VDSC) activity [1]; however, the clinical features of VDSC-inhibiting anticonvulsants cannot be fully understood solely from the VDSC inhibition profile. Indeed, LCM has a more favourable cognitive profile compared to CBZ and ZNS [2,3]. Both CBZ and ZNS enhance fast inactivation without affecting the slow inactivation phases of VDSC, whereas LCM enhances slow inactivation without affecting the fast inactivation phases [4]. These differences in the VDSC inhibition profiles among the three anticonvulsants may distinguish LCM from CBZ and ZNS in terms of cognitive function. Phenytoin and lamotrigine enhance both the slow and fast VDSC inactivation phases [4]; however, phenytoin and lamotrigine negatively and positively affect cognitive function, respectively [5]. Therefore, these clinical and preclinical findings suggest that the clinical spectrum of each anticonvulsant should be considered as being constituted by the integration between the inhibition of spreading epileptic hyperexcitability via VDSC inhibition with effects on other transmission regulating systems [1,6].

In the past two decades, molecular biological and pharmacological studies have gradually been revealing that the complex mechanisms of the development of epileptogenesis/ictogenesis comprise the acquisition of everlasting epilepsy-specific transmission abnormality induced by genetic abnormality with event-related transmission abnormality [7,8,9,10,11,12,13]. Recently, we have demonstrated the pathomechanism and pathophysiology of autosomal-dominant sleep-related hypermotor epilepsy (ADSHE) [14] with S284L mutation in the CHRNA4 gene encoding the α4 subunit of the nicotinic acetylcholine receptor (nAChR) [7,8,9,10]. ADSHE (previously known as autosomal-dominant nocturnal frontal lobe epilepsy) was first identified as a distinct familial idiopathic epilepsy in 1994 [15]. Although, typically, ADSHE seizures are symptomatically comparable to those seen in frontal lobe epilepsy and occur predominantly during non-rapid eye movement sleep [14,16,17], numerous missense mutations in various genes have been identified in many ADSHE pedigrees [14,17]. Usually, the majority of ADSHE seizures are well controlled by CBZ, which leads to remission in approximately 50–60% of ADSHE patients [16,18]; however, ADSHE patients with a CBZ-resistant feature require other anticonvulsants such as ZNS and LCM [17,18,19]. Furthermore, ADSHE seizures are the sole major symptom of ADSHE patients, and additional neuropsychiatric disturbances have been reported in lower than 3% of ADSHE patients [14]; however, ADSHE patients with an S284L-mutation have been known to be comorbid with a cognitive deficits such as schizophrenia-like psychosis, autism spectrum disorder, and intellectual disability [17,19].

Our recent studies using a genetic animal model with rats, namely S286L-TG, bearing the missense S286L-mutation of rat *Chrna4* that corresponds to an S284L-mutation in human *CHRNA4*, demonstrated several pathomechanisms and pathophysiologies of ADSHE seizures with an S284L-mutation [7,8,9,10]. Primarily, the impaired excitatory function of S286L-mutant α4β2-nAChR generates relatively GABAergic disinhibition in intrathalamic circuits, resulting in hyperexcitable glutamatergic transmission in both the thalamocortical motor and thalamic hyperdirect pathways [7,8,9,10]. Additionally, S286L-mutant α4β2-nAChR creates deficits in inhibitory regulation on connexin43 (Cx43) expression in the astroglial plasma membrane [8,9]. Based on these functional abnormalities, including the intrathalamic circuits, thalamocortical motor, thalamic hyperdirect pathways, and the physiological bursts, sleep spindle during non-rapid eye movement sleep possibly activate upregulated Cx43 hemichannel activity, leading to the generation of ADSHE focus in the frontal cortex [8]. We also demonstrated the three candidate pathophysiologies of CBZ-resistant/ZNS-sensitive ADSHE seizures with S284L-mutation. ZNS compensates the GABAergic disinhibition induced by impaired S286L-mutant α4β2-nAChR in the focus region [7]. ZNS inhibits the thalamic hyperdirect pathway by enhancing the endogenous group II metabotropic glutamate receptor, xanthurenic acid, resulting in the prevention of nocturnal paroxysmal dystonia, an ADSHE paroxysmal movement disorder [10,20]. Furthermore, chronic administration of ZNS prevents ADSHE focus generation due to reduction of astroglial Cx43 expression on the plasma membrane in the focus region [8].

Cx43 is the most predominant expression connexin isoform in astrocytes [21]. Six connexins assemble to form a homomeric/heteromeric connexon. Two connexon in two neighbouring cells (including neurons, astrocytes, oligodendrocytes, and microglia) form a gap-junction with an aqueous pore and charged surface walls, but a single connexon contributes to the chemical connection between the intracellular and extracellular spaces as a hemichannel [21]. Gap-junctions and hemichannels are crucial to the coordination/maintenance of physiologic activity, including neuronal excitability, synaptic plasticity, tripartite synaptic transmission, and homeostasis maintenance in the central nervous system [21]. Astroglial hemichannels also regulate ionic homeostasis, including ion movement regulation between intracellular and extracellular spaces, and the release of several gliotransmitters, including adenosine triphosphate (ATP), nicotinamide adenine dinucleotide, l-glutamate, or prostaglandins, involved in autocrine/paracrine signalling [22]. During the resting stage, astroglial hemichannels do not contribute to gliotransmitter release due to their low opening probability [22,23]; however, activation of hemichannel induced by plasma membrane depolarisation and extracellular/intracellular cation mobilization (i.e., elevations of extracellular K^+^ and intracellular Ca^2+^ with a reduction of extracellular Ca^2+^) generates gliotransmitter release through activated hemichannels [22,23]. It has also been well established that astrocytes play important roles in the development of epileptogenesis under subclinical pro-inflammatory responses [21,24]. Furthermore, Cx43 is involved in memory formation under physiological function, whereas both impaired and hyperactivated astroglial function generate cognitive disturbance [25].

The expression of Cx43 is increased in glia but not in neurons in pentylenetetrazole kindled rats [26], as well as in patients with refractory temporal lobe epilepsy and type IIB focal cortical dysplasia [27,28]. Furthermore, the upregulation of Cx43 in S286L-TG has also been demonstrated [8,9]. In particular, the inhibitors of connexin channels can prevent the onset of epileptic seizures [29]. These findings suggest that inhibition of the functional connexin expression in the plasma membrane with hemichannel activity can prevent the astroglial processes of epileptic seizures. Furthermore, we have already hypothesised that the interplay between astroglial depolarization and hemichannel activation produces a further astroglial regenerative activation circular sequence: plasma membrane depolarization activates an accelerated increase in the movements of cations via activated hemichannels, resulting in further depolarization of the astroglial membrane potential [8,9,30]. Therefore, astroglial hemichannel inhibition is a reasonable candidate for anticonvulsive medication; however, considering the effects of astroglial hemichannel inhibition on cognition [25] is important for ensuring the quality of life (QOL) of patients with epilepsy. We have also reported that upregulated Cx43 and/or astroglial hemichannels play important roles in the pathophysiology of CBZ-resistant and ZNS-sensitive epileptic seizures of S286L-TG [8,9]. Furthermore, several clinical studies have reported that LCM can suppress CBZ-resistant ADSHE seizures [18,19]. Therefore, we hypothesise that functional abnormality regarding astroglial hemichannels provides the pathophysiology of CBZ-resistant epileptic seizures. According to our hypothesis, in the present study, to explore the effects of VDSC-inhibiting anticonvulsants CBZ, LCM, and ZNS on astroglial hemichannel expression and activity, we determined the concentration-dependent effects of CBZ, LCM, and ZNS on Cx43 expression in the plasma membrane, and astroglial transmission-associated activated hemichannels using microdialysis, primary cultured astrocytes, and the Simple Western system.

## 2. Results

### 2.1. Effects of the Levels of Extracellular Ca^2+^ and K^+^ on the Astroglial Release of l-Glutamate and ATP (Study_1)

Electrophysiological study demonstrated that decreased levels of extracellular Ca^2+^ and increased extracellular K^+^ activate hemichannel activity [22,23,31]. To study gliotransmitter release through astroglial hemichannels, the interaction between extracellular cations and hemichannel inhibitors on the astroglial release of l-glutamate and ATP was determined using primary cultured astrocytes. To determine the effects of hemichannel inhibitors, carbenoxolone (CBX; a non-selective inhibitor) [30,32] and GAP19 (a selective Cx43 inhibitor) [30,32] on the astroglial release of l-glutamate and ATP induced by Ca^2+^-free (FC-ACSF), 100 mM K^+^ (HK-ACSF) or Ca^2+^-free with 100 mM K^+^ (FCHK-ACSF) containing artificial cerebrospinal fluid (ACSF), during the 21 days after culture (DIV21) to DIV28, the astrocytes were incubated in Dulbecco’s modified Eagle’s medium containing 10% fetal calf serum (fDMEM) without any agents. On DIV28, after being washed out, during pretreatment incubation, the astrocytes were incubated in ACSF with or without CBX (100 μM) or GAP19 (20 μM) [8,30] for 20 min. After pretreatment incubation, the astrocytes were incubated in FC-ACSF, HK-ACSF, or FCHK-ACSF containing the same agent of pretreatment for 20 min.

**Figure 1:**
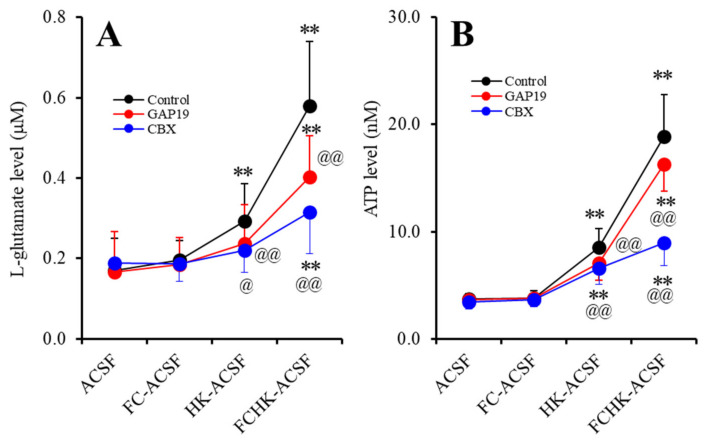
Effects of the extracellular Ca^2+^ and K^+^ and hemichannel inhibitors carbenoxolone (CBX; a non-selective inhibitor, 100 μM) and GAP19 (a selective Cx43 inhibitor, 20 μM) on the astroglial release of (**A**) l-glutamate and (**B**) adenosine triphosphate (ATP). Primary cultured astrocytes were incubated in artificial cerebrospinal fluid (ACSF), Ca^2+^-free ACSF (FC-ACSF), K^+^-containing ACSF (HK-ACSF; 100 mM), and Ca^2+^-free with 100 mM K^+^-containing ACSF (FCHK-ACSF) for 20 min. Ordinate: mean ± standard deviation (SD) (*n* = 6) of the extracellular levels of l-glutamate (μM) and ATP (nM). ** *p* < 0.01 relative to ACSF, and ^@^ *p* < 0.05 and ^@@^ *p* < 0.01 relative to the control (without hemichannel inhibitors) by MANOVA with Tukey’s post-hoc test.

Study_1 indicates the statistical significance regarding the interaction between the extracellular cation levels and the hemichannel inhibitors on the astroglial release of l-glutamate (F_ion_ (3,45) = 62.9, *p* < 0.01; F_agent_ (2,15) = 2.8, > 0.05; and F_ion*agent_ (6,45) = 7.2, *p* < 0.01) and ATP (F_ion_ (3,45) = 157.6, *p* < 0.01; F_agent_ (2,15) = 9.1, *p* < 0.01; and F_ion*agent_ (6,45) = 13.8, *p* < 0.01). Extracellular Ca^2+^-free (incubated in FC-ACSF for 20 min) did not affect the astroglial release of l-glutamate or ATP compared to those in ACSF (Figure 1A,B). Increased extracellular K^+^ (100 mM) (incubated in HK-ACSF for 20 min) enhanced the astroglial release of l-glutamate and ATP (Figure 1A,B). Extracellular Ca^2+^-free with 100 mM K^+^ condition (incubated in FCHK-ACSF for 20 min) drastically increased the astroglial release of l-glutamate and ATP (Figure 1A,B). Cx43 hemichannel inhibitor GAP19 (20 μM) and non-selective hemichannel inhibitor CBX (100 μM) suppressed the HK-ACSF- and FCHK-ACSF-evoked release of l-glutamate and ATP (Figure 1A,B). The results in Study_1 indicate that astroglial hemichannels are non-functional during resting stage. A decrease in extracellular Ca^2+^ alone cannot activate astroglial hemichannels but can accelerate the function of activated hemichannels by elevation of the level of extracellular K^+^.

### 2.2. Effects of the Hemichannel Inhibitors on the Repetitive FCHK-ASCF-Evoked Astroglial Release of l-Glutamate and ATP

It is well known that during the resting stage, hemichannels have a low opening probability, but under extracellular cation conditions, increased K^+^ and decreased Ca^2+^ levels activate hemichannel activity [22,23]. A previous microdialysis study demonstrated that repetitive 100 mM K^+^-evoked stimulation increased l-glutamate release via activated astroglial hemichannels in a use-dependent manner [8]. In a previous study using primary cultured astrocytes, 100 mM K^+^-evoked stimulation generated the activation of astroglial hemichannels, but 50 mM K^+^-evoked stimulation did not [30]. Therefore, to study the mechanisms of use-dependent repetitive K^+^-evoked gliotransmitter release, the effects of 20 μM GAP19 and 100 μM CBX on the astroglial release of l-glutamate and ATP induced by repetitive FCHK-ACSF-evoked stimulation were determined.

#### 2.2.1. Effects of GAP19 and Carbenoxolone (CBX) on the FCHK-ACSF-Evoked Astroglial Release of l-Glutamate and ATP through Activated Hemichannels (Study_2)

**Figure 2:**
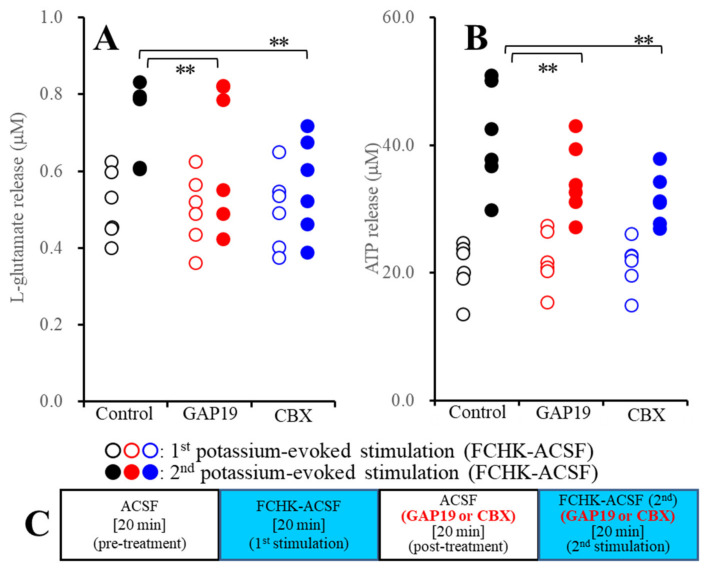
Effects of the hemichannel inhibitors (20 μM GAP19 and 100 μM CBX) on the astroglial release of (**A**) l-glutamate, (**B**) ATP induced by FCHK-ACSF associated with activated astroglial hemichannels, and (**C**) the experimental design. Ordinate: mean ± SD (*n* = 6) of the levels of l-glutamate (μM) and ATP (nM). ** *p* < 0.01 relative to the control by two-way ANOVA with Tukey’s post-hoc test.

To study the effects of the hemichannel inhibitors on gliotransmitter release through activated hemichannels, after the first FCHK-ACSF-evoked stimulation for 20 min, the astrocytes were incubated in ACSF containing 100 μM CBX or 20 μM GAP19 for 20 min (post-treatment). After the post-treatment, the astrocytes were incubated in FCHK-ACSF containing the same agent for 20 min again (the second FCHK-ACSF-evoked stimulation). The schematic experimental design of Study_2 is represented in Figure 2C.

Study_2 indicates the statistical significance regarding the interaction between the extracellular cation levels and the hemichannel inhibitors on the astroglial release of l-glutamate (F_event_ (2,20) = 13.3, *p* < 0.01; F_Cx43_ (1,10) = 4.9, *p* > 0.05; and F_event*Cx43_ (2,20) = 10.6, *p* < 0.01) and ATP (F_event_ (2,20) = 19.8, *p* < 0.01; F_Cx43_ (1,10) = 24.1, *p* < 0.01; and F_event*Cx43_ (2,20) = 27.3, *p* < 0.01). Both CBX and GAP19 inhibited the second FCHK-ACSF-evoked astroglial release of l-glutamate and ATP (Figure 2A,B). The results of Study_2 indicate that both CBX and GAP19 inhibit astroglial release through activated hemichannels.

#### 2.2.2. Effects of GAP19 and CBX on the Repetitive FCHK-ACSF-Evoked Astroglial Release of l-Glutamate and ATP through Unactivated and Activated Hemichannels (Study_3)

**Figure 3:**
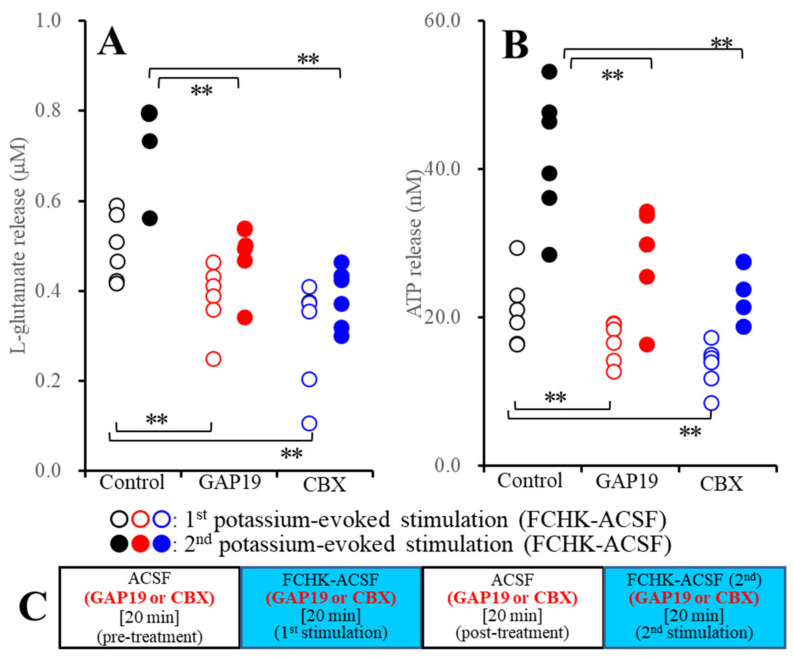
Effects of the hemichannel inhibitors (20 μM GAP19 and 100 μM CBX) on the repetitive K^+^-evoked (FCHK-ACSF) astroglial release of (**A**) l-glutamate, (**B**) ATP, and (**C**) the experimental design. Ordinate: mean ± SD (*n* = 6) of the levels of l-glutamate (μM) and ATP (nM). ** *p* < 0.01 relative to the control by two-way ANOVA with Tukey’s post-hoc test.

To study the effects of the hemichannel inhibitors on the activation-process of astroglial hemichannels, during Study_3, primary cultured astrocytes were incubated in ACSF or FCHK-ACSF without (control) or with 100 μM CBX or 20 μM GAP19. The schematic experimental design of Study_3 is represented in Figure 3C. Before the first stimulation, the astrocytes were incubated in ACSF containing 100 μM CBX or 20 μM GAP19 for 20 min (pretreatment), and after pretreatment, the astrocytes were evoked by FCHK-ACSF containing the same hemichannel inhibitor for 20 min (the first FCHK-ACSF stimulation). After the post-treatment in the same medium of pretreatment, the astrocytes were also incubated in FCHK-ACSF containing the same agent for 20 min again (the second FCHK-ACSF-evoked stimulation).

Study_3 indicates the statistical significance regarding the interaction between the extracellular cation levels and the hemichannel inhibitors on the astroglial release of l-glutamate (F_event_ (2,20) = 73.1, *p* < 0.01; F_Cx43_ (1,10) = 5.0, *p* < 0.05; and F_event*Cx43_ (2,20) = 11.7, *p* < 0.01) and ATP (F_event_ (2,20) = 88.3, *p* < 0.01; F_Cx43_ (1,10) = 22.7, *p* < 0.01; and F_event*Cx43_ (2,20) = 18.3, *p* < 0.01). After the application of 100 μM CBX and 20 μM GAP19 to unactivated hemichannels, both CBX and GAP19 reduced the first FCHK-ACSF-evoked release of l-glutamate and ATP (Figure 3A,B). Similar to the first FCHK-ACSF-evoked stimulation, both CBX and GAP19 reduced the second FCHK-ACSF-evoked release of l-glutamate and ATP (Figure 3A,B). Taken together with the results of Study_2, both CBX and GAP19 suppress not only the astroglial release through activated hemichannels, but also the activation process of the astroglial hemichannels.

### 2.3. Concentration-Dependent Effects of Carbamazepine (CBZ), Lacosamide (LCM), and Zonisamide (ZNS) on the Astroglial Release of l-Glutamate and ATP (Study_4)

To study the concentration-dependent effects of the subchronic administration of CBZ, LCM, and ZNS on the basal astroglial release of l-glutamate and ATP, during DIV21 to DIV28, the astrocytes were incubated in fDMEM containing CBZ (0, 10, 30, or 100 μM), LCM (0, 10, 30, or 100 μM) or ZNS (0, 30, 100, or 300 μM) for 7 days. On DIV28, after being washed out, during the pretreatment, the astrocytes were incubated in ACSF containing the same agent for 20 min. To study the concentration-dependent effects of the acute administration of CBZ, LCM, and ZNS on the basal astroglial release of l-glutamate and ATP, during DIV21 to DIV28, the astrocytes were incubated in fDMEM without anticonvulsant for 7 days. On DIV28, after being washed out, during the pretreatment, the astrocytes were incubated in ACSF with or without CBZ (10, 30, or 100 μM), LCM (10, 30, or 100 μM) or ZNS (30, 100, or 300 μM) for 20 min.

**Figure 4:**
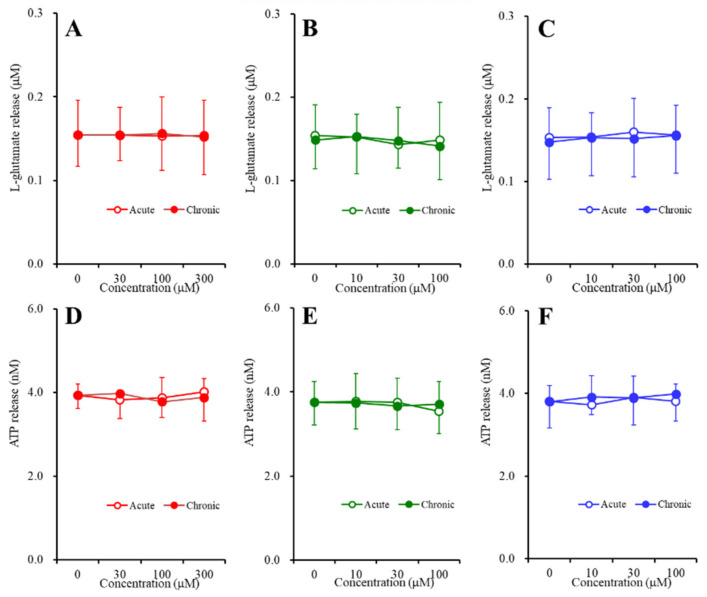
Concentration-dependent effects of the acute (open circles) and subchronic (closed circles) administration of (**A**,**D**) zonisamide (ZNS) (30, 100, or 300 μM), (**B**,**E**) carbamazepine (CBZ) (10, 30, or 100 μM), and (**C**,**F**) lacosamide (LCM) (10, 30, or 100 μM) on the basal astroglial release of (**A**–**C**) l-glutamate and (**D**–**F**)ATP. Ordinate: mean ± SD (*n* = 6) of the basal astroglial release of l-glutamate (μM) and ATP (nM). Abscissa: concentration of ZNS, CBZ, and LCM (μM).

Neither acute nor subchronic administration of CBZ (10, 30, or 100 μM), LCM (10, 30, or 100 μM), or ZNS (30, 100, or 300 μM) affected the basal astroglial release of l-glutamate and ATP (Figure 4).

**Figure 5:**
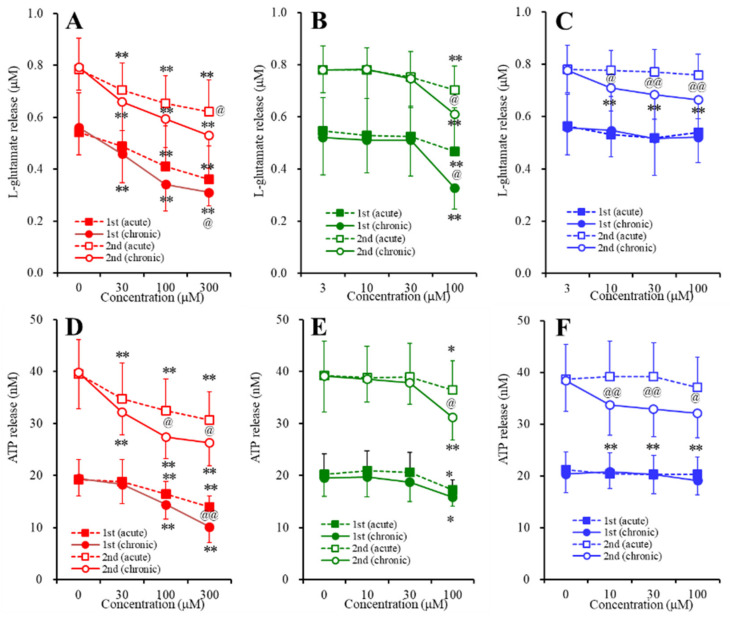
Concentration-dependent effects of the acute (squares) and subchronic (circles) administration of (**A**,**D**) ZNS (30, 100, or 300 μM), (**B**,**E**) CBZ (10, 30, or 100 μM), and (**C**,**F**) LCM (10, 30, or 100 μM) on the repetitive K^+^-evoked (first: closed marks; second: opened marks) astroglial release of (**A**–**C**) l-glutamate and (**D**–**F**) ATP. Ordinate: mean ± SD (*n* = 6) of the astroglial release of l-glutamate (μM) and ATP (nM). Abscissa: concentration of ZNS, CBZ, and LCM (μM). * *p* < 0.05 and ** *p* < 0.01 relative to the anticonvulsant-free condition, and ^@^ *p* < 0.05 and ^@@^ *p* < 0.01 relative to acute administration by MANOVA with Tukey’s multiple comparison. The F-values of the effects of ZNS on l-glutamate and ATP were F_ZNS_ (3,30) = 77.4 (*p* < 0.01), F_event_ (1,10) = 147.6 (*p* < 0.01), F_treatment_ (1,10) = 0.4 (*p* > 0.05), F_ZNS*treatment_ (3,30) = 3.2 (*p* < 0.05), F_event*treatment_ (1,10) = 0.2 (*p* > 0.05), F_ZNS*event_ (3,30) = 0.5 (*p* > 0.05), and F_ZNS*event*treatment_ (3,30) = 0.2 (*p* > 0.05), and on ATP were F_ZNS_ (3,30) = 167.9 (*p* < 0.01), F_event_ (1,10) = 422.5 (*p* < 0.01), F_treatment_ (1,10) = 0.8 (*p* > 0.05), F_ZNS*treatment_ (3,30) = 10.2 (*p* < 0.01), F_event*treatment_ (1,10) = 0.7 (*p* > 0.05), F_ZNS*event_ (3,30) = 19.1 (*p* < 0.01), and F_ZNS*event*treatment_ (3,30) = 1.3 (*p* > 0.05), respectively. The F-values of the effects of CBZ on l-glutamate and ATP were F_CBZ_ (3,30) = 64.3 (*p* < 0.01), F_event_ (1,10) = 402.2 (*p* < 0.01), F_treatment_ (1,10) = 0.4 (*p* > 0.05), F_CBZ*treatment_ (3,30) = 12.4 (*p* < 0.01), F_event*treatment_ (1,10) = 1.1 (*p* > 0.05), F_CBZ*event_ (3,30) = 0.5 (*p* > 0.05), and F_CBZ*event*treatment_ (3,30) = 0.2 (*p* > 0.05), and on ATP were F_CBZ_ (3,30) = 26.9 (*p* < 0.01), F_event_ (1,10) = 388.0 (*p* < 0.01), F_treatment_ (1,10) = 0.4 (*p* > 0.05), F_CBZ*treatment_ (3,30) = 8.7 (*p* > 0.05), F_event*treatment_ (1,10) = 0.1 (*p* > 0.05), F_CBZ*event_ (3,30) = 1.0 (*p* > 0.05), F_CBZ*event*treatment_ (3,30) = 1.8 (*p* > 0.05), respectively. The F-values of the effects of LCM on l-glutamate and ATP were F_LCM_ (3,30) = 9.4 *(p* < 0.01), F_event_ (1,10) = 286.3 (*p* < 0.01), F_treatment_ (1,10) = 0.3 (*p* > 0.05), F_LCM*treatment_ (3,30) = 2.4 (*p* > 0.05), F_event*treatment_ (1,10) = 6.4 (*p* < 0.01), F_LCM*event_ (3,30) = 1.3 (*p* > 0.05), and F_LCM*event*treatment_ (3,30) = 2.3 (*p* > 0.05), and on ATP were F_LCM_ (3,30) = 11.3 (*p* < 0.01), F_event_ (1,10) = 441.7 (*p* < 0.01), F_treatment_ (1,10) = 0.8 (*p* > 0.05), F_LCM*treatment_ (3,30) = 3.9 (*p* < 0.05), F_event*treatment_(1,10) = 6.6 (*p* < 0.05), F_LCM*event_ (3,30) = 5.5 (*p* < 0.01), and F_LCM*event*treatment_ (3,30) = 8.8 (*p* < 0.01), respectively.

To study the effects of the acute and subchronic administration of CBZ, LCM, and ZNS on the astroglial release of l-glutamate and ATP induced by repetitive FCHK-ACSF-evoked stimulation, after the above sampling of the basal release, the astrocytes were incubated in FCHK-ACSF containing the same agent for 20 min (the first stimulation). After the first stimulation, the incubation medium was switched to ACSF containing the same agent (post-treatment) for 20 min, and then the astrocytes were evoked by FCHK-ACSF containing the same agent for 20 min (the second stimulation) again. The F-values by MANOVA are indicated in the legend of Figure 5.

The acute and subchronic administration of ZNS (30, 100, or 300 μM) inhibited the astroglial release of both l-glutamate and ATP induced by the first and second FCHK-ACSF-evoked stimulations in a concentration-dependent manner (Figure 5A,D). CBZ (10, 30, or 100 μM) also inhibited the astroglial release of both l-glutamate and ATP induced by the first and second FCHK-ACSF-evoked stimulations (Figure 5C,F). The acute and subchronic administration of therapeutic-relevant concentration of CBZ (10 or 30 μM) did not affect the repetitive FCHK-ACSF-evoked release of l-glutamate and ATP; however, the acute and subchronic administration of a supratherapeutic concentration of CBZ (100 μM) inhibited the repetitive FCHK-ACSF-evoked astroglial release of l-glutamate and ATP (Figure 5B,E). LCM (10, 30, or 100 μM) also inhibited the astroglial release of both l-glutamate and ATP induced by the first and second FCHK-ACSF-evoked stimulations (Figure 5C,F). The acute administration of LCM (10, 30, or 100 μM) did not affect the repetitive FCHK-ACSF-evoked astroglial release of l-glutamate and ATP, whereas the subchronic administration of LCM (10, 30, or 100 μM) inhibited the repetitive FCHK-ACSF-evoked astroglial release of l-glutamate and ATP (Figure 5C,F).

Therefore, both the acute and the subchronic administration of therapeutic-relevant or supratherapeutic concentration of CBZ, LCM, and ZNS did not affect the astroglial release associated with unactivated hemichannels; however, CBZ, LCM, and ZNS exhibited distinct targets regarding the astroglial release associated with hemichannels.

### 2.4. Concentration-Dependent Effects of the Subchronic Administration of LCM, CBZ, and ZNS on Cx43 Expression in the Plasma Membrane Fraction of Primary Cultured Astrocytes (Study_4)

The Cx43 expression of hepatic and cardiac cells can be decreased by 2% dimethyl sulfoxide and 4% ethanol, respectively [33,34]. In the present study, the levels of dimethyl sulfoxide and ethanol in fDMEM for the subchronic administration of 100 μM CBZ and 100 μM LCM were 0.2% and 1%, respectively. The effects of 0.2% dimethyl sulfoxide and 1% ethanol on Cx43 expression in the plasma membrane fraction of primary cultured astrocytes could not be observed (Supplementary Figure S1).

**Figure 6:**
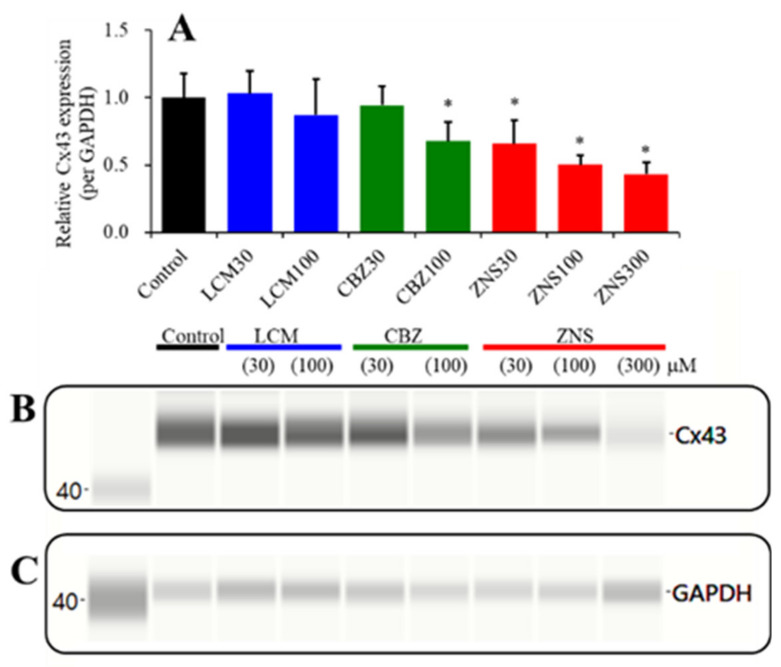
Concentration-dependent effects of the subchronic administration of LCM (30 or 100 μM), CBZ (30 or 100 μM), and ZNS (30, 100, or 300 μM) on the Cx43 expression in the plasma membrane fraction of primary cultured astrocytes (**A**) and pseudo-gel images using the Simple Western results with anti-Cx43 (**B**) and anti-GAPDH (**C**) antibodies for blotting of the plasma membrane fractions. In (**A**), ordinate: mean ± SD (*n* = 6) of the relative protein level of Cx43. * *p* < 0.05 relative to the control by one-way ANOVA with Tukey’s multiple comparison.

The subchronic administration (for 7 days) of both a therapeutic-relevant (30 or 100 μM) and a supratherapeutic (300 μM) concentration of ZNS decreased the Cx43 expression in the plasma membrane fraction of primary cultured astrocytes in a concentration-dependent manner (F (3,20) = 20.2, *p* < 0.01) (Figure 6). A therapeutic-relevant concentration of CBZ (30 μM) did not affect Cx43 expression, but a supratherapeutic concentration of CBZ (100 μM) decreased the Cx43 expression in the plasma membrane (F (2,15) = 7.3, *p* < 0.01) (Figure 6). Neither a therapeutic-relevant (30 μM) nor a supratherapeutic (100 μM) concentration of LCM affected the Cx43 expression in the plasma membrane (Figure 6).

### 2.5. Effects of the Extracellular K^+^ and Ca^2+^ Levels on the Release of l-Glutamate and ATP in the Orbitofrontal Cortex (OFC)

To study of the regulation mechanisms of the extracellular cations on the release of l-glutamate and ATP, the present study determined the effects of perfusion with modified Ringer’s solution (MRS) containing 50 mM K^+^ (MK-MRS), 100 mM K^+^ (HK-MRS), and Ca^2+^-free with 100 mM K^+^ (FCHK-MRS) on the extracellular levels of l-glutamate and ATP in the OFC using microdialysis. The perfusion medium was commenced with MRS or MRS containing 0.1 μM PSB12379 (ectoATPase inhibitor) for l-glutamate and ATP, respectively. When the coefficients of variation for l-glutamate and ATP reached <5% over a period of 60 min (stabilization), control data were obtained over another 60 min period (pretreatment period), and then the perfusion medium was switched to MK-MRS, HK-MRS, or FCHK-MRS for 20 min.

**Figure 7:**
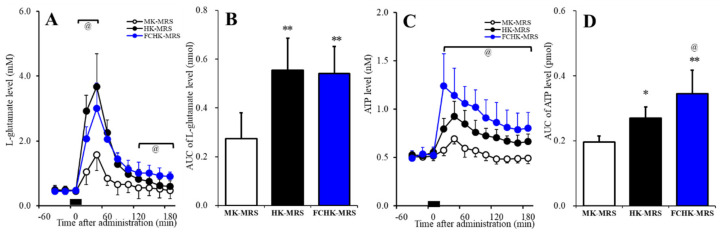
Effects of perfusion with 50 mM K^+^ containing modified Ringer’s solution (MRS) (MK-MRS), 100 mM K^+^ containing MRS (HK-MRS), and Ca^2+^-free with 100 mM K^+^ containing MRS (FCHK-MRS) on the extracellular levels of (**A**,**B**) l-glutamate and (C,D) ATP in the orbitofrontal cortex (OFC). Ordinates: the mean extracellular levels of l-glutamate (μM) and ATP (nM) (*n* = 6); abscissas: time after perfusion with MK-MRS, HK-MRS, or FCHK-MRS for 20 min (black bars). The area under the curve (AUC) values of the extracellular levels of l-glutamate (nmol) and ATP (pmol) after perfusion with MK-MRS, HK-MRS, or FCHK-MRS during 20–180 min of (**A**,**B**) are represented in (**C**,**D**), respectively. * *p* < 0.05 and ** *p* < 0.01 relative to MK-MRS, and ^@^ *p* < 0.05 relative to HK-MRS by MANOVA with Tukey’s post-hoc test.

The extracellular l-glutamate levels in the OFC were increased by the extracellular K^+^ levels in a concentration-dependent manner (F_Time_ (1.4,21.3) = 209.5, *p* < 0.01); F_ion_ (2,15) = 10.4, *p* < 0.01; and F_Time*ion_ (2.8,21.3) = 21.3, *p* < 0.01) (Figure 7A,B). The area under curve (AUC) values of the l-glutamate release induced by HK-MRS and FCHK-MRS were almost equal (Figure 7B); however, the l-glutamate release induced by FCHK-MRS (depletion of Ca^2+^) decreased in the initial phase (20–40 min), but increased in the late phase (120–180 min) of the l-glutamate release compared to the HK-MRS-evoked release (Figure 7A). The extracellular ATP levels in the OFC were increased by the extracellular K^+^ levels in a concentration-dependent manner (F_Time_ (3.2,48.5) = 67.7, *p* < 0.01; F_ion_ (2,15) = 14.0, *p* < 0.01; and F_Time*ion_ (6.5,48.5) = 11.4, *p* < 0.01) (Figure 7C,D). Furthermore, depletion of extracellular Ca^2+^ enhanced the K^+^-evoked ATP release (Figure 7C,D).

### 2.6. Effects of the Subchronic Administration of a Therapeutic-Relevant Dose of CBZ, LCM, and ZNS on the Repetitive FCHK-MRS-Evoked Release of l-Glutamate and ATP in the OFC

The results using primary cultured astrocytes (Study_1~Study_4) indicate that repetitive FCHK-ACSF-evoked stimulation is a candidate astroglial activation technique. Furthermore, in our previous study, local administration of ZNS and the Cx43 selective inhibitor GAP19 prevented the repetitive HK-MRS-evoked l-glutamate release through activated astroglial hemichannels in the secondary motor cortex [8]. Based on the results of our previous study and Study_1~Study_4 in this report, to study the effects of the subchronic administration of a therapeutic-relevant dose of ZNS, CBZ, and LCM on the release of l-glutamate and ATP in the OFC, rats were subchronically administrated ZNS, CBZ, and LCM (0 or 25 mg/kg/day for 7 days) using a subcutaneous osmotic pump (2ML_1, Alzet) [9].

**Figure 8:**
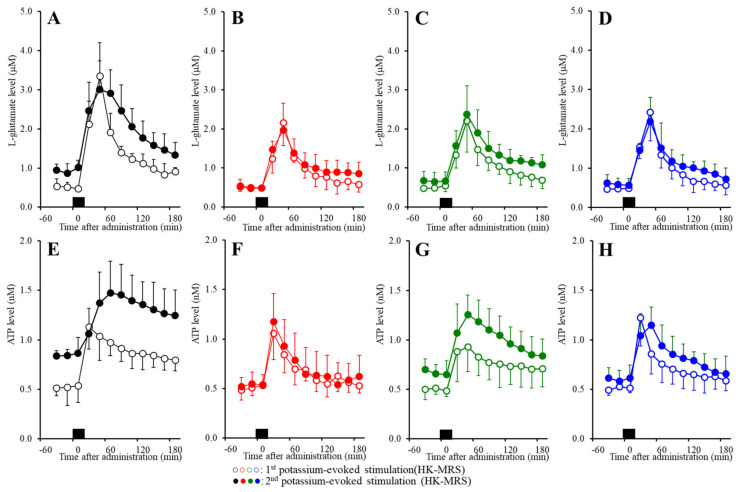
Effects of the subchronic administration of a therapeutic-relevant dose of (**B**,**F**) ZNS, (**C**,**G**) CBZ, and (**D**,**H**) and LCM (25 mg/kg/day for 7 days) on the repetitive K^+^-evoked (first stimulation: opened circles; second stimulation: closed circles) release of (**A**–**D**) l-glutamate and (**E**–**H**) ATP in the OFC. Rats were subchronically administrated with a therapeutic-relevant dose of anticonvulsants. Perfusion medium in the OFC was commenced with MRS. After the stabilization of the levels of l-glutamate and ATP in the OFC, the perfusion medium was switched from MRS to FCHK-MRS for 20 min (first stimulation). After the first K^+^-evoked stimulation, the perfusate was returned to MRS for 240 min (recovery). Following recovery, the perfusate was switched to FCHK-MRS for 20 min again (second stimulation). After the second FCHK-MRS stimulation, the perfusate was returned to MRS again. Ordinates: mean extracellular levels of l-glutamate (μM) and ATP (nM) (*n* = 6); abscissas: time after the first or second FCHK-MRS stimulations (min). Black bars indicate the perfusion with FCHK-MRS (first and second K^+^-evoked stimulation).

**Figure 9:**
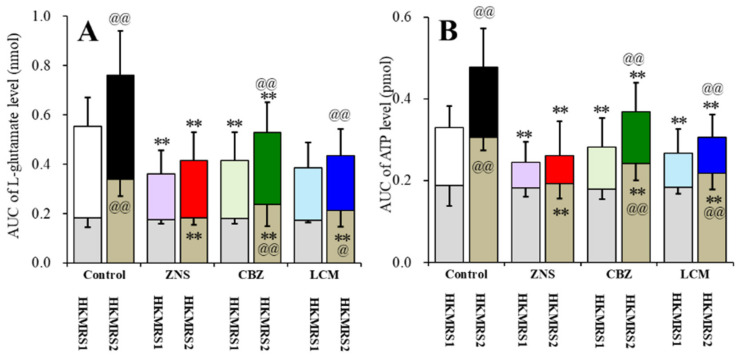
Effects of the subchronic administration of a therapeutic-relevant dose of ZNS, CBZ, and LCM (25 mg/kg/day for 7 days) on the repetitive FCHK-MRS-evoked (first stimulation: HKMRS1; second stimulation: HKMRS2) release of (**A**) l-glutamate and (**B**) ATP in the OFC. Ordinates: mean AUC values of the extracellular levels of l-glutamate (nmol) and ATP (pmol) before (basal extracellular l-glutamate levels) and after K^+^-evoked stimulation (from 20 to 180 min) of Figure 1. Gray and colored columns indicate the AUC values of the basal and K^+^-evoked releases, respectively. * *p* < 0.05 and ** *p* < 0.01; relative to the control, ^@^ *p* < 0.05 and ^@@^ *p* < 0.01 relative to HKMRS (first simulation) by MANOVA with Tukey’s post-hoc test. The F-values of the effects of ZNS on l-glutamate and ATP were F_ZNS_ (1,200) = 185.0 (*p* < 0.01), F_time_ (9,200) = 53.4 (*p* < 0.01), F_event_ (1,10) = 47.0 (*p* < 0.01), F_ZNS*time_ (9,200) = 3.7 (*p* < 0.01), F_ZNS*event_(1,200) = 16.9 (*p* < 0.01), F_event*time_ (9,200) = 2.3 *(p* < 0.05), and F_ZNS*event*time_ (9,200) = 1.1 (*p* > 0.05), and on ATP were F_ZNS_ (1,200) = 216.2 (*p* < 0.01), F_time_ (9,200) = 11.5 (*p* < 0.01), F_event_ (1,10) = 70.5 (*p* < 0.01), F_ZNS*time_ (9,200) = 4.8 (*p* < 0.01), F_ZNS*event_ (1,200) = 45.7 (*p* < 0.01), F_event*time_ (9,200) = 0.9 (*p* > 0.05), and F_ZNS*event*time_ (9,200) = 1.7 (*p* > 0.05), respectively. The F-values of the effects of CBZ on l-glutamate and ATP were F_CBZ_ (1,200) = 76.9 (*p* < 0.01), F_time_ (9,200) = 50.4 (*p* < 0.01), F_event_ (1,10) = 63.2 (*p* < 0.01), F_CBZ*time_ (9,200) = 2.5 (*p* < 0.05), F_CBZ*event_ (1,200) = 5.7 (*p* < 0.05), F_event*time_ (9,200) = 1.8 (*p* > 0.05), and F_CBZ*event*time_ (9,200) = 1.0 (*p* > 0.05), and on ATP were F_CBZ_ (1,200) = 57.6 (*p* < 0.01), F_time_ (9,200) = 11.4 (*p* < 0.01), F_event_ (1,10) = 138.4 (*p* < 0.01), F_CBZ*time_ (9,200) = 0.6 (*p* > 0.05), F_CBZ*event_ (1,200) = 9.6 (*p* < 0.05), F_event*time_ (9,200) = 1.7 (*p* > 0.05), F_CBZ*event*time_ (9,200) = 1.1 (*p* > 0.05), respectively. The F-values of the effects of LCM on l-glutamate and ATP were F_LCM_ (1,200) = 147.8 (*p* < 0.01), F_time_ (9,200) = 58.0 (*p* < 0.01), F_event_ (1,10) = 44.2 (*p* < 0.01), F_LCM*time_ (9,200) = 2.2 (*p* < 0.05), F_LCM*event_ (1,200) = 16.5 (*p* < 0.01), F_event*time_ (9,200) = 2.7 (*p* < 0.01), and F_CBZ*event*time_ (9,200) = 0.8 (*p* > 0.05), and on ATP were F_LCM_ (1,200) = 148.3 (*p* < 0.01), F_time_ (9,200) = 13.8 (*p* < 0.01), F_event_ (1,10) = 103.5 (*p* < 0.01), F_LCM*time_ (9,200) = 3.8 (*p* < 0.05), F_LCM*event_ (1,200) = 35.6 *(p* < 0.01), F_event*time_ (9,200) = 3.2 (*p* < 0.01), and F_LCM*event*time_ (9,200) = 0.7 (*p* > 0.05), respectively.

Repetitive FCHK-MRS stimulation increased the extracellular levels of l-glutamate and ATP (Figure 8A,E and Figure 9A,B) in the OFC in a use-dependent manner. After the 240 min recovery from the first FCHK-MRS-evoked stimulation, the basal extracellular levels of l-glutamate and ATP were higher than the basal levels of l-glutamate and ATP before the first FCHK-MRS stimulation (first vs. second in Figure 8A,E and Figure 9A,B). The second FCHK-MRS-evoked stimulation also increased the release of l-glutamate and ATP (Figure 8A,E and Figure 9A,B). The release of l-glutamate and ATP induced by the second FCHK-MRS-evoked stimulation was larger than those by the first FCHK-MRS-evoked stimulation (Figure 8A,E and Figure 9A,B).

Subchronic administration of a therapeutic-relevant dose of ZNS (25 mg/kg/day) for 7 days inhibited the repetitive FCHK-MRS-evoked release of l-glutamate and ATP in the OFC (Figure 8B,F and Figure 9A,B). The differences of the basal extracellular levels of l-glutamate and ATP before and after the first FCHK-MRS-evoked stimulation could not be detected in rats administrated ZNS subchronically (Figure 8B,F and Figure 9A,B). The differences between the release of l-glutamate and ATP induced by the first and the second FCHK-MRS-evoked stimulations also could not be detected in rats administrated ZNS subchronically (Figure 8B,F and Figure 9A,B).

Subchronic administration of a therapeutic-relevant dose of CBZ (25 mg/kg/day) for 7 days inhibited the release of both l-glutamate and ATP induced by repetitive FCHK-MRS-evoked stimulation in the OFC (Figure 8C and Figure 9A,B). Subchronic CBZ administration decreased the elevation of the basal levels of l-glutamate and ATP after the first FCHK-MRS-evoked stimulation compared to the control rats, whereas the basal extracellular levels of l-glutamate and ATP after the second FCHK-MRS-evoked stimulation became larger than those before the first stimulation (Figure 8C,G and Figure 9A,B).

Subchronic administration of a therapeutic-relevant dose of LCM (25 mg/kg/day) for 7 days inhibited the release of l-glutamate and ATP induced by repetitive FCHK-MRS-evoked stimulation in the OFC (Figure 8D,H and Figure 9A,B). Subchronic LCM administration decreased the elevation of the basal levels of l-glutamate and ATP after the first FCHK-MRS-evoked stimulation compared to the control rats, whereas the basal extracellular levels of l-glutamate and ATP after the first FCHK-MRS-evoked stimulation became larger than those before the first stimulation (Figure 8D,H and Figure 9A,B).

## 3. Discussion

### 3.1. Regulation Mechanisms of the Astroglial Gliotransmitter Release Associated with Cx43-Containing Hemichannels

The present study demonstrated various components of event-related astroglial gliotransmitter release and their specific regulation mechanisms using in vivo microdialysis and in vitro primary cultured astrocytes. It has been established that neurotransmitter exocytosis is generated by the Ca^2+^-dependent exocytosis process, which is activated by elevation of the Ca^2+^ levels in the presynaptic active zone via inflow through the voltage-sensitive Ca^2+^ channel or ionotropic glutamate receptor, or output from the endoplasmic reticulum [6,35,36]. In the present study, during the resting stage, astroglial hemichannels had an essentially low opening probability similar to previous reports [8,22,23,30], since the hemichannel inhibitors CBX and GAP19 did not affect the astroglial release of l-glutamate and ATP during incubation in ACSF (Figure 1). Contrary to neurotransmitter exocytosis, astroglial hemichannel opening requires depolarization, increased extracellular K^+^, and/or decreased extracellular Ca^2+^ [8,22,23,30]. In the present study, reduced extracellular Ca^2+^ alone (FC-ACSF) did not affect astroglial release, but accelerated astroglial release was induced by increased extracellular K^+^ (FCHK-ACSF), which was suppressed by the hemichannel inhibitors, CBX and GAP19, contrary to basal release (Figure 1). Therefore, depletion of extracellular Ca^2+^ does not act as a generator, but as an accelerator for astroglial release through activated hemichannels. Taken together with previous electrophysiological studies, the potential for activation of hemichannel activity is probably as follows: depolarization > elevation of extracellular K^+^ > depletion of extracellular Ca^2+^.

It is well known that elevation of the levels of extracellular K^+^ generates depolarization; however, the sensitivity of neurotransmission and gliotransmission to extracellular K^+^ is different. Indeed, neurotransmitter exocytosis is activated by 25 mM K^+^-evoked stimulation, whereas astroglial release is not increased by 50 mM K^+^-evoked stimulation [30,37]. This discrepancy of K^+^ sensitivity between neurotransmission and gliotransmission is caused by the lower VDSC expression density in non-epileptic astrocytes [38]. Taken together with the previous findings, the present results can lead to the candidate hypothesis for transmission abnormalities between the neurotransmission and gliotransmission associated with activated hemichannel. Toxic hyperexcitable events such as epileptic seizures generate an elevation of extracellular K^+^ and a depletion of extracellular Ca^2+^ [39,40]. The depletion of the levels of extracellular Ca^2+^ suppresses the propagation of epileptic hyperexcitability in attenuation of neurotransmission [39]; however, at the same time, the combination of astroglial depolarization with extracellular ionic abnormality induced by epileptic hyperexcitability activates astroglial hemichannels [39]. The present results support the transition of major components for excitability propagation from neurotransmission to gliotransmission during the sustained hyperexcitable stage. Study of both microdialysis and primary cultured astrocytes demonstrated that repetitive FCHK-MRS-evoked and FCHK-ACSF-evoked stimulations increase the release of l-glutamate and ATP through activated hemichannels in a use-dependent manner (Figure 2, Figure 3, and Figure 9). Therefore, during the initial phase of an epileptic seizure, epileptic hyperexcitability is propagated by Ca^2+^-dependent neurotransmitter exocytosis, whereas during the late phase, the major propagation components probably shift from neurotransmission to astroglial transmission due to extracellular ionic abnormality. Furthermore, the activation state of astroglial hemichannels is persistent over several hours [8], and the gliotransmitter release through astroglial hemichannels is enhanced and persistent in a use-dependent manner (Figure 2 and Figure 3). These prolonged and use-dependent hyperexcitabilities associated with astroglial transmission contribute to the development of event-related ictogenesis [8,13,17].

### 3.2. Inhibitory Effects of the VDSC-Inhibiting Acticonvulsants CBZ, LCM, and ZNS on Activated Cx43 Hemichannels

We have already demonstrated that the inhibitory effects of ZNS on the Cx43 expression in the plasma membrane fraction play important roles in the inhibition of CBZ-resistant partial epileptic seizures of the genetic ADSHE model S286L-TG [8]. In the present study, astroglial hemichannels are suggested to be activated by toxic hyperexcitable depolarization and its induced depletion of extracellular Ca^2+^ with elevation of the level of extracellular K^+^. Based on these findings, before experiment, we speculated that CBZ prevented the repetitive FCHK-MRS-evoked release of l-glutamate and ATP using in vivo microdialysis, but did not affect the repetitive FCHK-ACSF-evoked astroglial release of l-glutamate and ATP using primary cultured astrocytes. The results in this study were in partial agreement with our expectations but could also suggest the more complex mechanisms of ZNS, CBZ, and LCM.

In the microdialysis study, the subchronic administration of therapeutic-relevant dose of CBZ, LCM, and ZNS reduced the repetitive FCHK-MRS-evoked release of l-glutamate and ATP compared to the control (Figure 8 and Figure 9). The use-dependent potentiation of the repetitive FCHK-MRS-evoked release was abolished by ZNS, but was observed in rats subchronically administrated CBZ and LCM (Figure 8B,F and Figure 9A,B). Furthermore, elevation of the basal extracellular levels of l-glutamate and ATP after FCHK-MRS-evoked stimulation, which are released through activated hemichannels [8], was observed in CBZ- and LCM-administrated rats (Figure 8B,F and Figure 9A,B). These results suggest that subchronic administration of therapeutic-relevant dose of CBZ and LCM can prevent propagation of epileptic discharges via their VDSC inhibition but cannot completely prevent activation of astroglial hemichannels. This discrepancy between CBZ, LCM, and ZNS in terms of the repetitive FCHK-MRS-evoked release is induced by two possibilities, a dose-dependent factor or a direct effect on astroglial hemichannels, including activity and hemichannel expression.

In order to clarify the mechanisms of the discrepancy between CBZ, LCM, and ZNS, we determined the acute and subchronic effects of the three anticonvulsants on astroglial hemichannel activity, as well as the subchronic effects of the three anticonvulsants on the Cx43 expression in the astroglial plasma membrane using primary cultured astrocytes. During the resting stage (incubation in ACSF), the astroglial release of l-glutamate and ATP was not affected by acute or subchronic administration of CBZ, LCM, or ZNS (Figure 4). ZNS acutely and subchronically inhibited the first and second FCHK-ACSF-evoked astroglial release in a concentration-dependent manner, but subchronic ZNS predominantly inhibited this release compared to acute administration (Figure 5A,D), possibly caused by reduced Cx43 expression in the plasma membrane (Figure 6). Thus, ZNS acutely inhibits astroglial hemichannel activation directly, with a subchronic reduction of functional hemichannels. Contrary to ZNS, neither acute nor subchronic administration of therapeutic-relevant concentration of CBZ (10 or 30 μM) affected the repetitive FCHK-ACSF-evoked astroglial release, whereas both acute and subchronic administration of supratherapeutic concentration of CBZ (100 μM) could inhibit it (Figure 5B,E). Furthermore, the inhibitory effects of subchronic administration of supratherapeutic CBZ concentration were predominant compared to acute administration. Indeed, the Cx43 expression in the plasma membrane was decreased and was not affected by supratherapeutic and therapeutic-relevant concentration of CBZ, respectively (Figure 6). Thus, therapeutic-relevant dose/concentration of CBZ could not suppress activation of astroglial hemichannels directly. These differences between ZNS and CBZ can explain the pathophysiology of CBZ-resistant/ZNS-sensitive seizures of S286L-TG [7,8,10]. In our previous study, we speculated that the mechanisms of ZNS-specific effects of astroglial hemichannels are modulated by the enhancement of ubiquitin ligase, the metabotropic glutamate receptor, or inhibition of carbonic anhydrase [7,8,10,20,41,42]. In order to clarify the specific mechanism of the effects of ZNS on astroglial hemichannel expression in the plasma membrane, we shall demonstrate in further studies in the future.

Subchronic LCM administration reduced the repetitive FCHK-ACSF-evoked astroglial release in a concentration-dependent manner without affecting Cx43 expression, but acute administration did not affect the repetitive FCHK-ACSF-evoked astroglial release (Figure 5C,F and Figure 6). The mechanisms of the inhibitory effects of LCM on the astroglial release associated with hemichannels are different from those of ZNS but can be evaluated to be within the same targets. Inhibition of collapsin response mediator protein 2 (CRMP2) is considered to be a candidate target regarding the antiepileptic action of LCM, in addition to VDSC inhibition [43]. CRMP2 is a positive mediator against VDSC, the voltage-sensitive Ca^2+^ channel, and neurite outgrowth [43]. The interaction between LCM or CRMP2 and connexin remains to be clarified, but LCM might inhibit the astroglial hemichannel activation process via CRMP2. Recently, the effectiveness of LCM on CBZ-resistant ADSHE seizures with CHRNA4 mutation was reported [18]. In other words, subchronic LCM administration directly suppresses the function of activated astroglial hemichannel activity without affecting hemichannel expression. Based on the effects of ZNS and LCM on astroglial hemichannels demonstrated in this study, the suppressive action of gliotransmission associated with astroglial hemichannels leads to novel reasonable add-on medication strategies.

Finally, the present study demonstrated the distinct effects between CBZ, LCM, and ZNS on astroglial transmission associated with activated hemichannels; however, this study has several limitations. Firstly, the concentration-dependent effects of the subchronic administration of CBZ, LCM, and ZNS on the Cx43 expression in the plasma membrane fraction of primary cultured astrocytes was demonstrated, but connexin26 and connexin30 are also expressed in astrocytes [21]. Unfortunately, we could not obtain suitable antibodies of connexin26 and connexin30 for the Simple Western system. We should have screened the antibodies of connexin26 and connexin30 to clarify the effects of the anticonvulsant on expressions of connexin26 and connexin30 in astrocytes. Secondly, the present study indicated that repetitive stimulation using FCHK-MRS and FCHK-ACSF is a pharmacologically useful technique for the activation of astroglial hemichannels. During repetitive and prolonged hyperexcitability, such as epileptic discharge, the levels of extracellular K^+^ increase and the levels of extracellular Ca^2+^ decrease; however, FCHK-ACSF and FCHK-MRS are more drastic conditions compared to the pathological toxic hyperactivation state. To clarify the pathomechanisms of epileptic seizures associated with extracellular cation mobilization, we should conduct a more detailed study to determine the concentration-dependent effects of the levels of extracellular Ca^2+^ and K^+^ in the lower range using epileptic animal models.

## 4. Materials and Methods

### 4.1. Experimental Animals

The animal care procedures, experimental procedures, and protocols for animal experiments were approved by the Animal Research Ethics Committee of the Mie University School of Medicine (No. 24–35-R3). All studies involving animals have been reported in accordance with the ARRIVE guidelines for reporting experiments involving animals [44]. A total of 66 adult rats were used in the microdialysis study, and 30 neonatal rats were used for the primary cultured astrocytes studies.

### 4.2. Preparation of the Primary Cultured Astrocytes

The astrocytes were prepared using a protocol adapted from previously described methods [20,30,35,45]. Pregnant Sprague-Dawley rats (SLC, Sizuoka, Japan), which were housed individually in cages, were kept in air-conditioned rooms (temperature, 22 ± 2 °C) set to a 12 h light/dark cycle, with free access to food and water. The cultured astrocytes were prepared from cortical astrocyte cultures of neonatal Sprague-Dawley rats (*n* = 30) sacrificed by decapitation at 0–24 h of age [24,45]. The cerebral hemispheres were removed under a dissecting microscope. Tissue was chopped into fine pieces using scissors, and then triturated briefly with a micropipette. The suspension was filtered using 70 µm nylon mesh (BD, Franklin Lakes, NJ, USA), and then centrifuged. The pellets were then re-suspended in 10 mL Dulbecco’s modified Eagle’s medium containing 10% fetal calf serum (fDMEM), which was repeated three times. After culture for 14 days (DIV14), the contaminated cells were removed by shaking in a standard incubator for 16 h at 200 rpm. On DIV21, the astrocytes were removed from the flasks by trypsinization, and then seeded directly onto translucent polyethylene terephthalate (PET) membrane (1.0 μm) with 24-well plates (BD) at a density of 1 × 10^5^ cells/cm^2^ for the experiments. From DIV21 to DIV28, the culture medium (fDMEM) was changed twice a week, and CBZ (0, 10, 30, or 100 μM containing 0.2% dimethyl sulfoxide as a vehicle), LCM (0, 10, 30, or 100 μM, containing 1% ethanol as a vehicle), or ZNS (0, 30, 100, or 300 μM) was added for subchronic administration (for 7 days).

On DIV28, the cultured astrocytes were washed out using artificial cerebrospinal fluid (ACSF), and this was repeated three times. The ACSF comprised NaCl 150.0 mM, KCl 3.0 mM, CaCl_2_ 1.4 mM, MgCl_2_ 0.8 mM, and glucose 5.5 mM, buffered to pH 7.3 with 20 mM HEPES buffer [30]. To study the effects of a decrease in the level of extracellular Ca^2+^ (Ca^2+^-free), an increase in the level of K^+^ (K^+^-evoked stimulation), and both complex conditions (Ca^2+^-free with K^+^-evoked stimulation) on the astroglial release of l-glutamate and ATP, the cultured astrocytes were incubated in Ca^2+^-free ACSF (FC-ACSF), ACSF containing 100 mM K^+^ (HK-ACSF), or Ca^2+^-free with 100 mM K^+^ ion ACSF (FCHK-ACSF) for 20 min [30]. The ionic composition was modified, and isotonicity was maintained by an equimolar decrease of sodium ions [9,30].

### 4.3. Simple Western Analysis

Simple Western analyses were performed using Wes (ProteinSimple, Santa Clara, CA, USA) according to the ProteinSimple user manual [7,30]. The lysate of the primary cultured astrocytes was mixed with a master mix (ProteinSimple) to a final concentration of 1× sample buffer, 1× fluorescent molecular weight markers, and 40 mM dithiothreitol, and then heated at 95 °C for 5 min. The samples, blocking reagent, primary antibodies, horseradish peroxidase (HRP)-conjugated second antibodies, chemiluminescent substrate, and separation and stacking matrices were also dispensed to designated wells in a 25-well plate. After plate loading, the separation electrophoresis and immunodetection steps took place in the capillary system and were fully automated. The Simple Western analyses were carried out at room temperature, and the instrument’s default settings were used. Capillaries were first filled with the separation matrix, followed by the stacking matrix and about 40 nL sample loading. During electrophoresis, the proteins were separated on the basis of molecular weight through the stacking and separation matrices at 250 volts for 40–50 min, and then immobilized on the capillary wall using proprietary photo-activated capture chemistry. The matrices were then washed out. The capillaries were next incubated with a blocking reagent for 15 min, and the target proteins were immunoprobed with primary antibodies followed by HRP-conjugated secondary antibodies (Anti-Rabbit Detection Module, DM-001, ProteinSimple). The antibodies of GAPDH (NB300-322SS, Novus Biologicals, Littleton, CO, USA) and Cx43 (C6219, Sigma-Aldrich, St. Louis, MO, USA) were diluted in antibody diluent (ProteinSimple) at a 1:100 dilution. The antibody incubation time was 0–120 min with antibody diluents. Luminol and peroxide (ProteinSimple) were then added to generate chemiluminescence, which was captured by a charge-coupled device (CCD) camera. The digital image was analyzed with Compass software (ProteinSimple), and the quantified data of the detected protein were reported as molecular weight and signal/peak intensity.

### 4.4. Preparation of the Microdialysis System

Sprague-Dawley rats (SLC, Shizuoka, Japan) were housed in individual cages in air-conditioned rooms (temperature 22 ± 2 °C) with a 12 h light/dark cycle and ad libitum access to food and water. After the subchronic administration of 25 mg/kg/day CBZ, LCM, and ZNS for 7 days using a subcutaneously osmotic pump (2ML_1, Alzet, Cupertino, CA), the rats were anesthetized with 1.8% isoflurane and then placed in a stereotactic frame [46,47,48]. A concentric direct insertion-type dialysis probe (0.22 mm diameter, 3 mm exposed membrane; Eicom, Kyoto, Japan) was implanted in the orbitofrontal cortex (OFC: A = +3.2 mm, L = +2.4 mm, V = −6.5 mm, relative to bregma) [49,50].

Perfusion experiments were started 18 h after recovery from isoflurane anesthesia. The perfusion rate was set at 2 μL/min in all experiments using modified Ringer’s solution (MRS) composed of the following (in mM): NaCl (145.0), KCl (2.7), CaCl_2_ (1.2), and MgCl_2_ (1.0), buffered with 2 mM phosphate buffer and 1.1 mM Tris buffer at pH 7.4. To determine the effects of the levels of extracellular K^+^ and Ca^2+^, the perfusion medium was changed from MRS to 50 mM K^+^ containing MRS (MK-MRS): NaCl (97.7), KCl (50.0), CaCl_2_ (1.2), and MgCl_2_ (1.0); 100 mM K^+^ containing MRS (HK-MRS): NaCl (47.7), KCl (100.0), CaCl_2_ (1.2), and MgCl_2_ (1.0); and Ca^2+^-free with 100 mM K^+^ containing MRS (FCHK-MRS): NaCl (49.1), KCl (100.0), and MgCl_2_ (1.0), buffered with 2 mM phosphate buffer and 1.1 mM Tris buffer at pH 7.4 for 20 min [51,52,53]. To determine the levels of extracellular l-glutamate and ATP, MRS with and without 0.1 μM PSB12379 (ectoATPase inhibitor) was perfused, respectively. When the coefficients of variation for l-glutamate and ATP reached <5% over a period of 60 min (stabilization), control data were obtained over another 60 min period (pretreatment period), followed by switching to MK-MRS, HK-MRS, or FCHK-MRS for 20 min.

To explore the effects of the subchronic administration of a therapeutic-relevant dose of CBZ, LCM, and ZNS on astroglial hemichannel-associated transmitter release in the OFC induced by repetitive FCHK-MRS-evoked stimulations, the perfusion medium was commenced with MRS. After stabilization of the levels of l-glutamate and ATP, the perfusate was switched to FCHK-MRS for 20 min (the first FCHK-MRS-evoked stimulation). After the first stimulation, the perfusate was returned to MRS for 240 min (recovery). After the recovery, the perfusate was switched to FCHK-MRS (the second stimulation) for 20 min. After the second stimulation, the perfusate was returned to MRS again.

Following the microdialysis experiments, the rats were anesthetized under 1.8% isoflurane, and their brains were removed. The locations of the dialysis probes were verified by histological examination using 200-μm-thick tissue slices (Vibratome 1000, Technical Products International, St. Louis, MO).

### 4.5. Ultra-High-Performance Liquid Chromatography (UHPLC)

The level of l-glutamate was determined using ultra-high-performance liquid chromatography (UHPLC) equipped with xLC3185PU (Jasco, Tokyo, Japan) and fluorescence detection (xLC3120FP, Jasco), following dual derivatization with isobutyryl-l-cysteine and *o*-phthalaldehyde [54,55]. The derivatization solutions were prepared by dissolving isobutyryl-l-cysteine (2 mg) and o-phthalaldehyde (2 mg) in 0.1 mL ethanol, followed by the addition of 0.9 mL sodium borate buffer (0.2 M, pH 9.0) [35]. Automated pre-column derivatization was performed by drawing 5 μL aliquots of the sample, standard, or blank solutions and 5 μL of the derivatization solution together into a reaction vial, and then incubating for 5 min before injection. The derivatized samples (5 μL aliquots) were injected by an autosampler (xLC3059AS, Jasco). The analytical column (YMC Triat C18, particle 1.8 μm, 50 × 2.1 mm; YMC, Kyoto, Japan) was maintained at 45 °C and the flow rate was set at 500 μL/min. A linear gradient elution program was performed over a period of 10 min with mobile phases A (0.05 M citrate buffer, pH 5.0) and B (0.05 M citrate buffer containing 30% acetonitrile and 30% methanol, pH 3.5). The excitation/emission wavelengths of the fluorescence detector were set at 280/455 nm [56].

### 4.6. Ultra-High-Performance Liquid Chromatography with Mass Spectrometry (LCMS)

The levels of ATP were determined by UHPLC (ACQUITY UPLC H-Class system; Waters, Milford, MA, USA) with mass spectrometry (Acquity SQ detector; Waters). Twenty microliters of filtrated samples were injected using an autosampler (ACQUITY UPLC Sample Manager FTN; Waters). The concentrations of ATP were separated by UHPLC equipped with a Hypercarb column (particle 3 μm, 150 × 2.1 mm; Thermo, Waltham, MA, USA) at 35 °C, and the mobile phase was set at 450 µL/min. A linear gradient elution program was performed over 10 min with mobile phases A (1 mM ammonium acetate buffer, pH 11) and B (acetonitrile). The nitrogen flows of the desolvation and cone were set at 750 and 5 L/h, respectively, and the desolvation temperature was set at 450 °C. The cone voltage for the determination of ATP (m/z = 508.2) was 34 V.

### 4.7. Chemical Agents

The non-selective hemichannel inhibitor carbenoxolone (CBX) [30,32] and the selective Cx43 inhibitor GAP19 [30,32] were obtained from Funakoshi (Tokyo, Japan). The ecto-5’-nucleotidase (ectoATPase) inhibitor N6-Benzyl-α,β-methyleneadenosine 5’-diphosphate disodium salt (PSB12379) was obtained from Namiki (Tokyo, Japan). Lacosamide (LCM) was also obtained from Funakoshi. Carbamazepine (CBZ) was obtained from Tokyo Chemical Industry (Tokyo, Japan). Zonisamide sodium salt (ZNS) was provided by Dainippon-Sumitomo Pharma (Osaka, Japan).

All compounds were prepared on the day of the experiment. CBZ was initially dissolved at 50 mM in dimethyl sulfoxide; he final concentration of dimethyl sulfoxide was lower than 0.2% (*v/v*). LCM was initially dissolved at 10 mM in ethanol; the final concentration of ethanol was lower than 1.0% (*v/v*). GAP19, CBX, PSB12379, and ZNS were directly dissolved in ACSF or fDMEM for the primary cultured astrocyte study.

The therapeutic-relevant concentrations of CBZ, ZNS, and LCM were 17–42 μM [57,58,59], 47–330 μM [36,60,61], and 20–40 μM [4,62], respectively. Therefore, in the present study, the primary cultured astrocytes were subchronically administrated by CBZ (10, 30, or 100 μM), LCM (10, 30, or 100 μM), and ZNS (30, 100, or 300 μM) for 7 days, respectively. According to previous study, the rats were systemically administrated 25 mg/kg/day CBZ, LCM, and ZNS for 7 days [4,57,58,63,64,65] using a subcutaneously osmotic pump (2ML_1, Alzet); the nominal pumping rate and duration were 10 μL/h and 7 days.

### 4.8. Data Analysis

All experiments in this study were designed with equally sized animal groups (*n* = 6) without carrying out a formal power analysis, in keeping with previous studies [7,10,13,30,46,50,66]. All values are expressed as mean ± standard deviation (SD), and *p* < 0.05 (two-tailed) was considered statistically significant for all tests. The drug levels in the acute local and subchronic administrations were selected based on the values in previous studies [7,10,13,30,46,50,66,67]. Where possible, we sought to randomize and blind the data. In particular, for the determination of the extracellular transmitter levels, the sample order on the autosampler was determined by a random number table.

Data in the microdialysis study were analysed by the Mauchly’s sphericity test, followed by multivariate analysis of variance (MANOVA) using BellCurve for Excel ver. 3.2 (Social Survey Research Information Co., Ltd., Tokyo, Japan). When the data did not violate the assumption of sphericity (*p* > 0.05), the *f*-value of the MANOVA was analysed using sphericity-assumed degrees of freedom. However, if the assumption of sphericity was violated (*p* < 0.05), the *f*-value was analysed using Chi-Muller’s corrected degrees of freedom. When the F-value for the event/drug/time factors of the MANOVA was significant, the data were analysed by the Tukey’s post-hoc test. Data in the astroglial release studies were analysed by using MANOVA or analysis of variance (ANOVA) using BellCurve for Excel ver. 3.2 (Social Survey Research Information Co., Ltd., Tokyo, Japan). If the *f*-value for the event/drug/time factors of the MANOVA or ANOVA was significant, the data were analysed by Tukey’s multiple comparison. Data in the Simple Western study were analyzed by one-way analysis of variance (ANOVA) with Tukey’s multiple comparison using BellCurve for Excel. All statistical analyses complied with the recommendations on the experimental design and analysis in pharmacology [68].

### 4.9. Nomenclature of Targets and Ligands

The key protein targets and ligands in this article are hyperlinked to the corresponding entries in http://www.guidetopharmacology.org, which is the common portal for data from the IUPHAR/BPS Guide to PHARMACOLOGY [69], and are permanently archived in the Concise Guide to PHARMACOLOGY 2017/18 [70].

## 5. Conclusions

The present study demonstrated the distinct effects of VDSC-inhibiting anticonvulsants CBZ, LCM, and ZNS on gliotransmitter (i.e., l-glutamate and ATP) release through activated astroglial hemichannels. Astroglial hemichannels cannot release gliotransmission at low opening probability during the resting stage; however, plasma membrane depolarization and elevation of extracellular K^+^ with a reduction of extracellular Ca^2+^ activate astroglial hemichannels, and also generate persistent gliotransmitter release through activated hemichannels. A therapeutic-relevant dose/concentration of CBZ cannot affect astroglial hemichannel activity or Cx43 expression in the plasma membrane. Contrary to CBZ, both ZNS and LCM suppress gliotransmitter release through astroglial hemichannels, but the suppressive mechanisms between ZNS and LCM are distinct. A therapeutic-relevant dose/concentration of ZNS directly inhibits astroglial hemichannel activity with Cx43 expression in the plasma membrane in a concentration-dependent manner, whereas LCM inhibits hemichannel activity without affecting Cx43 expression. Therefore, the present study demonstrated the distinct pharmacological profiles between CBZ, LCM, and ZNS in terms of astroglial transmission associated with activated hemichannels. These results suggest that the distinct effects of these three VDSC-inhibiting anticonvulsants possibly contribute to the mechanisms of their antiepileptic profiles and/or adverse effects.

## Supplementary Materials

The following are available online at https://www.mdpi.com/1424-8247/13/6/117/s1, Figure S1: Effects of subchronic administration of dimethyl sulfoxide and ethanol on Cx43 expression in the plasma membrane fraction of primary cultured astrocytes (Study_4).
