# Supplementary material for: Effects of Carbamazepine, Lacosamide and Zonisamide on Gliotransmitter Release Associated with Activated Astroglial Hemichannels
Source: Pharmaceuticals (Basel). 2020 Jun 5;13(6):117. doi: 10.3390/ph13060117 (PMC7345221; doi:10.3390/ph13060117)
Supplement: Supplementary file 1 [file pharmaceuticals-13-00117-s001.pdf]

pharmaceuticals

MDPI

Supplemental data

Effects of subchronic administration of dimethyl sulfoxide and ethanol on Cx43 expression in the plasma membrane fraction of primary cultured astrocytes (Study_4)

The subchronic administration (for 7 days, from DIV21 to DIV28) of 0.2% (vol/vol) dimethyl sulfoxide (DMSO) and 1% (vol/vol) ethanol (EOH) did not affect the Cx43 expression in the plasma membrane fraction of primary cultured astrocytes using student T-test.

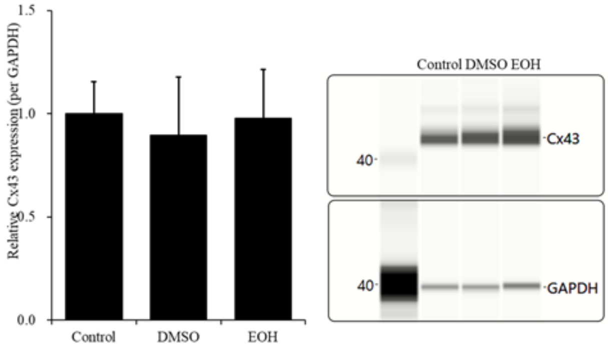

Figure. Effects of subchronic administration of dimethyl sulfoxide (DSMO) and ethanol (EOH) on Cx43 expression in the plasma membrane fraction of primary cultured astrocytes and pseudo-gel images using the Simple Western results with anti-Cx43 and anti-GAPDH antibodies for blotting of the plasma membrane fractions. Ordinate: mean ± SD (n = 6) of the relative protein level of Cx43.
